# Aberrant Scrotal Extension of an Incarcerated Incisional Hernia With Testicular Displacement

**DOI:** 10.7759/cureus.113450

**Published:** 2026-07-27

**Authors:** Armon Moradian, Patrick Martin, Claudia Pedreira, Kian Memari, Josue P Boutros, Robert Comperatore, Nuria Lawson, Michael Choi

**Affiliations:** 1 Medicine, Dr. Kiran C. Patel College of Osteopathic Medicine, Nova Southeastern University, Fort Lauderdale, USA; 2 Family Medicine, Dr. Kiran C. Patel College of Osteopathic Medicine, Nova Southeastern University, Fort Lauderdale, USA; 3 Family Medicine, Palmetto General Hospital, Hialeah, USA; 4 Surgery, Palmetto General Hospital, Hialeah, USA; 5 General Surgery, Palmetto General Hospital, Hialeah, USA; 6 Bariatric Surgery, Broward Health Imperial Point, Fort Lauderdale, USA

**Keywords:** abdominal wall reconstruction, aberrant hernia tract, incarcerated hernia, incisional hernia, scrotal hernia, testicular displacement, ventral hernia

## Abstract

Incisional hernias are common postoperative complications, whereas scrotal extension is usually associated with inguinal hernias traversing the inguinal canal. Scrotal involvement from a ventral incisional defect through a subcutaneous route superficial to the inguinal canal is uncommon and can substantially distort groin and testicular anatomy. We report a 62-year-old man with a remote laparoscopic right inguinal hernia repair who presented with acute pain, nausea, emesis, and a large non-reducible ventral and right inguinoscrotal swelling. Computed tomography demonstrated a midline/right paramedian ventral fascial defect containing small bowel and omentum, with inferior extension toward the right hemiscrotum and inferior displacement of the right testis. Scrotal Doppler ultrasonography confirmed preserved testicular perfusion. Diagnostic laparoscopy was converted to open exploration because of dense adhesions and distorted anatomy. The following two separate defects were identified: a large ventral/incisional defect and a right indirect inguinal defect. The scrotal component originated from the ventral hernia and coursed through a subcutaneous plane superficial to the inguinal canal. The right testis, which had been inferiorly displaced on imaging 48 h earlier, was found in a suprapubic position intraoperatively. The bowel was viable, the testis and spermatic cord structures were preserved, and both defects were repaired. This case emphasizes that complex ventral hernias may violate expected anatomical planes and that preoperative imaging should be integrated with, rather than substituted for, careful operative exploration.

## Introduction

Incisional hernias arise at sites of prior abdominal-wall disruption and occur after approximately 5%-20% of abdominal operations, depending on incision type, closure technique, wound complications, patient factors, and follow-up duration [1,2]. Ventral and incisional hernia repair also represents a substantial healthcare burden in the United States [3], and incisional hernias may adversely affect physical functioning, body image, and health-related quality of life [4]. Although most incisional hernias follow predictable fascial planes, their anatomy may be altered by chronicity, recurrent mechanical loading, adhesions, and local tissue remodeling [1,5].

Scrotal herniation most commonly occurs through the deep inguinal ring, inguinal canal, and superficial ring in an indirect inguinal hernia [5,6]. Complex scrotal inguinal hernias may increase the risk of seroma, bleeding, recurrence, spermatic cord injury, testicular ischemia, and testicular atrophy [6]. Cross-sectional imaging is therefore valuable when a hernia is incarcerated, recurrent, very large, or associated with groin or scrotal distortion because it can define defect size, contents, direction of extension, and relationships to adjacent structures [7].

Published literature describes giant inguinoscrotal hernias and an incisional hernia containing a bladder diverticulum that extended to the base of the scrotum [8,9]. However, a focused search did not identify a closely analogous report of a bowel-containing midline ventral incisional hernia traveling through a subcutaneous plane superficial to the inguinal canal with an interval change in testicular position. We present this apparently rare anatomical configuration and emphasize the operative implications of discordance between imaging and surgical findings.

## Case presentation

A 62-year-old man presented in September 2025 with acute abdominal and right groin pain radiating to the lower back and right lower extremity, accompanied by nausea and one episode of emesis. He reported recurrent right groin bulging that had previously reduced spontaneously. His medical history was notable for severe heart failure with reduced ejection fraction of 15%-20%. Eight years earlier, he had undergone laparoscopic right inguinal hernia repair for a direct inguinal hernia. The available prior records did not specify the trocar configuration, mesh type, or whether the current midline defect corresponded to a previous port site. No prior surgical-site infection, fascial dehiscence, or wound-healing complication was documented. Trocar-site incisional hernia is a recognized delayed complication of laparoscopic surgery, but the precise relationship between the prior repair and the present ventral defect could not be established [10].

Examination demonstrated an approximately 30×20 cm combined ventral and right inguinoscrotal swelling that was tense, tender, and non-reducible. The overlying abdominal and scrotal skin showed no erythema, warmth, discoloration, ulceration, or necrosis. The abdomen was soft and mildly distended, with normal bowel sounds and no guarding or rebound tenderness. The patient had passed stool the previous day. The right hemiscrotum was enlarged and tender, and the right testis could not be palpated separately; the left testis was palpable in its expected position. Concern for incarceration was based on acute worsening pain, persistent non-reducibility, nausea and emesis, enlargement of a previously intermittently reducible bulge, and bowel within the hernia on imaging. There were no clinical findings of peritonitis or advanced strangulation.

Laboratory testing demonstrated mild leukocytosis with a white blood cell count of 12.5×10³/µL and a normal lactate of 1.6 mmol/L (Table 1). C-reactive protein, erythrocyte sedimentation rate, and B-type natriuretic peptide were not obtained. Electrocardiography showed normal sinus rhythm. A transthoracic echocardiogram obtained two weeks before admission showed a dilated left ventricle with an ejection fraction of 15%-20%, functional mitral regurgitation, and elevated estimated left-sided filling pressures. Heart failure was considered an important anesthetic and perioperative risk factor, but it was not interpreted as the cause of the unusual hernia trajectory.

**Table 1:** Pertinent laboratory findings on presentation.

Test	Result	Reference range
White blood cell count (×10³/µL)	12.5	4.0-11.0
Hemoglobin (g/dL)	16.0	13.5-17.5
Hematocrit (%)	48	41-53
Platelet count (×10³/µL)	288	150-450
Sodium (mmol/L)	142	135-145
Potassium (mmol/L)	4.2	3.5-5.1
Creatinine (mg/dL)	0.9	0.7-1.3
Lactate (mmol/L)	1.6	0.5-2.2
Prothrombin time (s)	12	11-13.5
International normalized ratio	1.0	0.8-1.1

**Figure 1:**
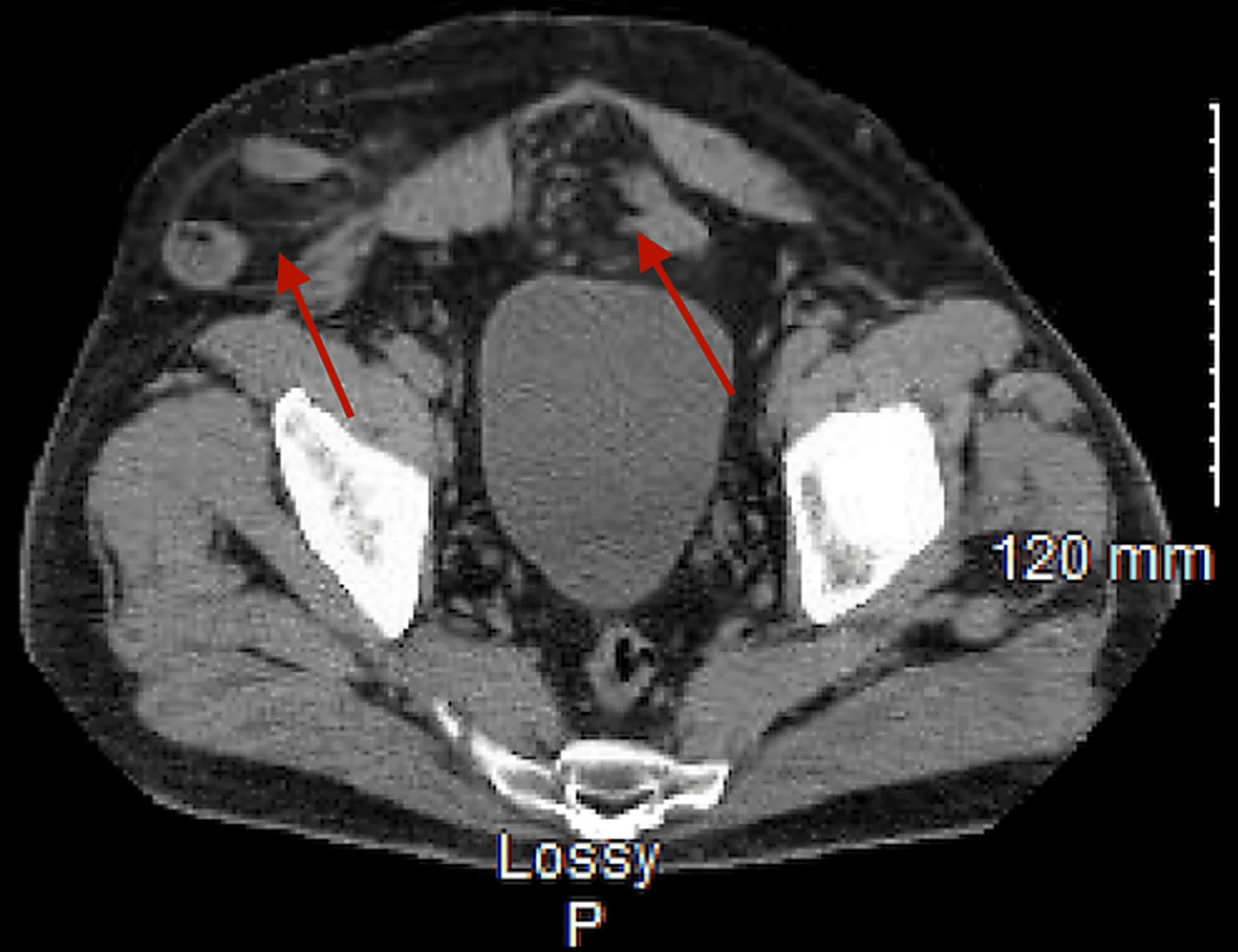
Ventral incisional hernia containing bowel and omentum. Axial CT demonstrates the midline and right paramedian ventral hernia containing small bowel and omentum (red arrows). The fascial defect measured 8.7×10.9 cm, and the hernia sac measured approximately 28.6×16.4×14.8 cm.

**Figure 2:**
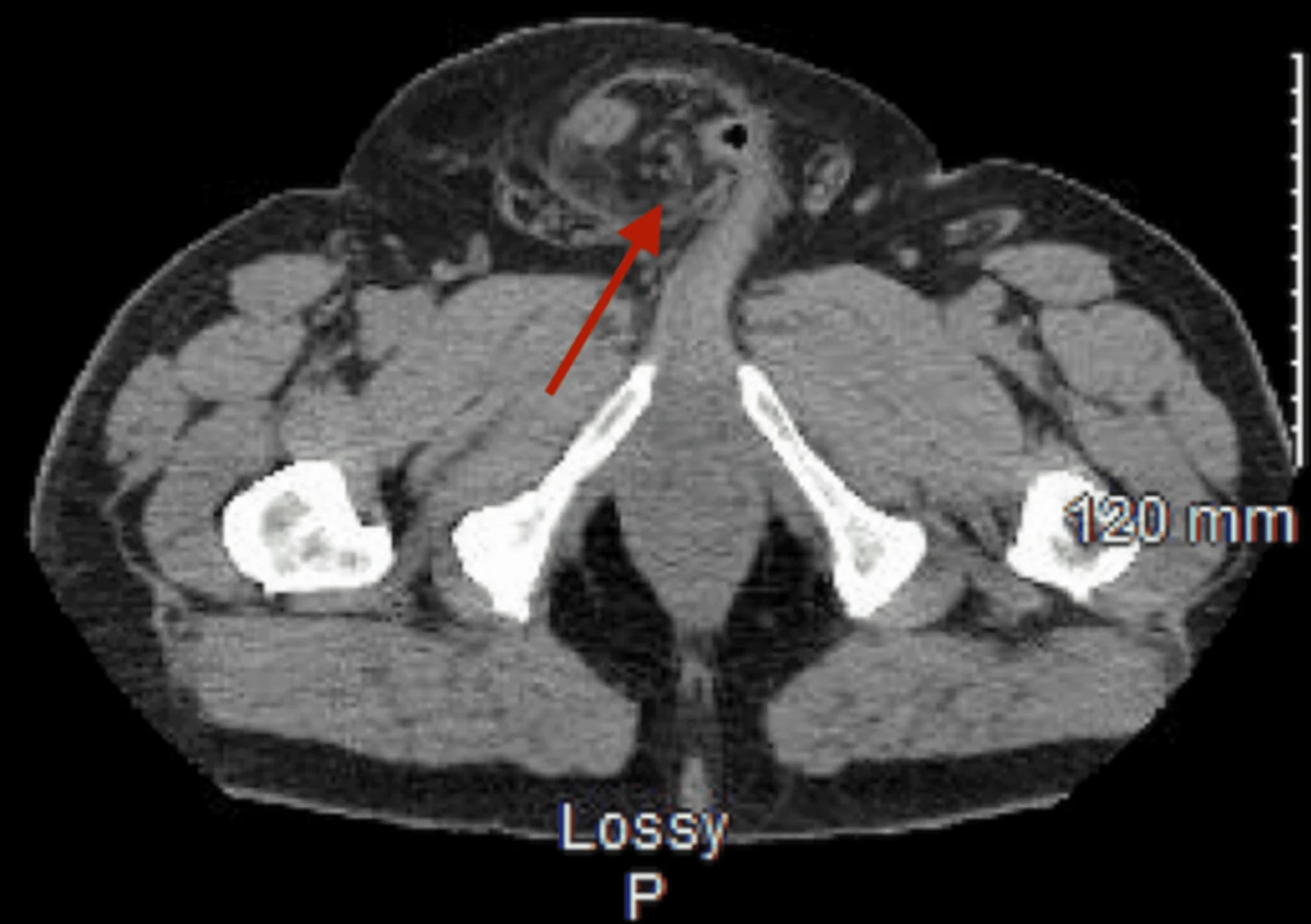
Inferior extension toward the right hemiscrotum. CT demonstrates inferior extension of the ventral hernia along the right lower abdominal wall toward the right hemiscrotum (red arrow).

**Figure 3:**
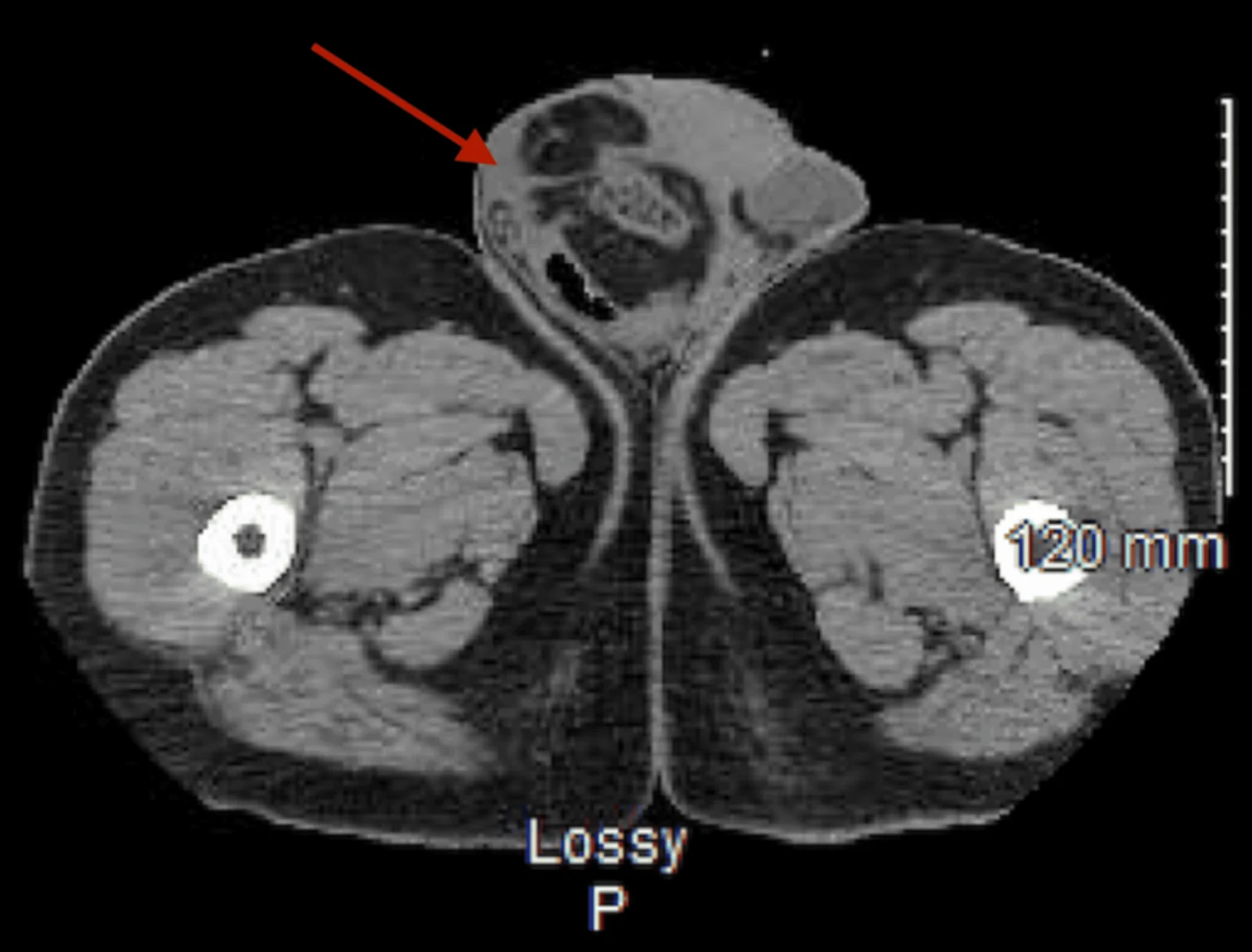
Apparent extension along the right inguinal region. Preoperative CT suggests extension toward the right hemiscrotum along the inguinal region (red arrow). Operative exploration subsequently demonstrated that the ventral component traveled through a subcutaneous plane superficial to the inguinal canal.

**Figure 4:**
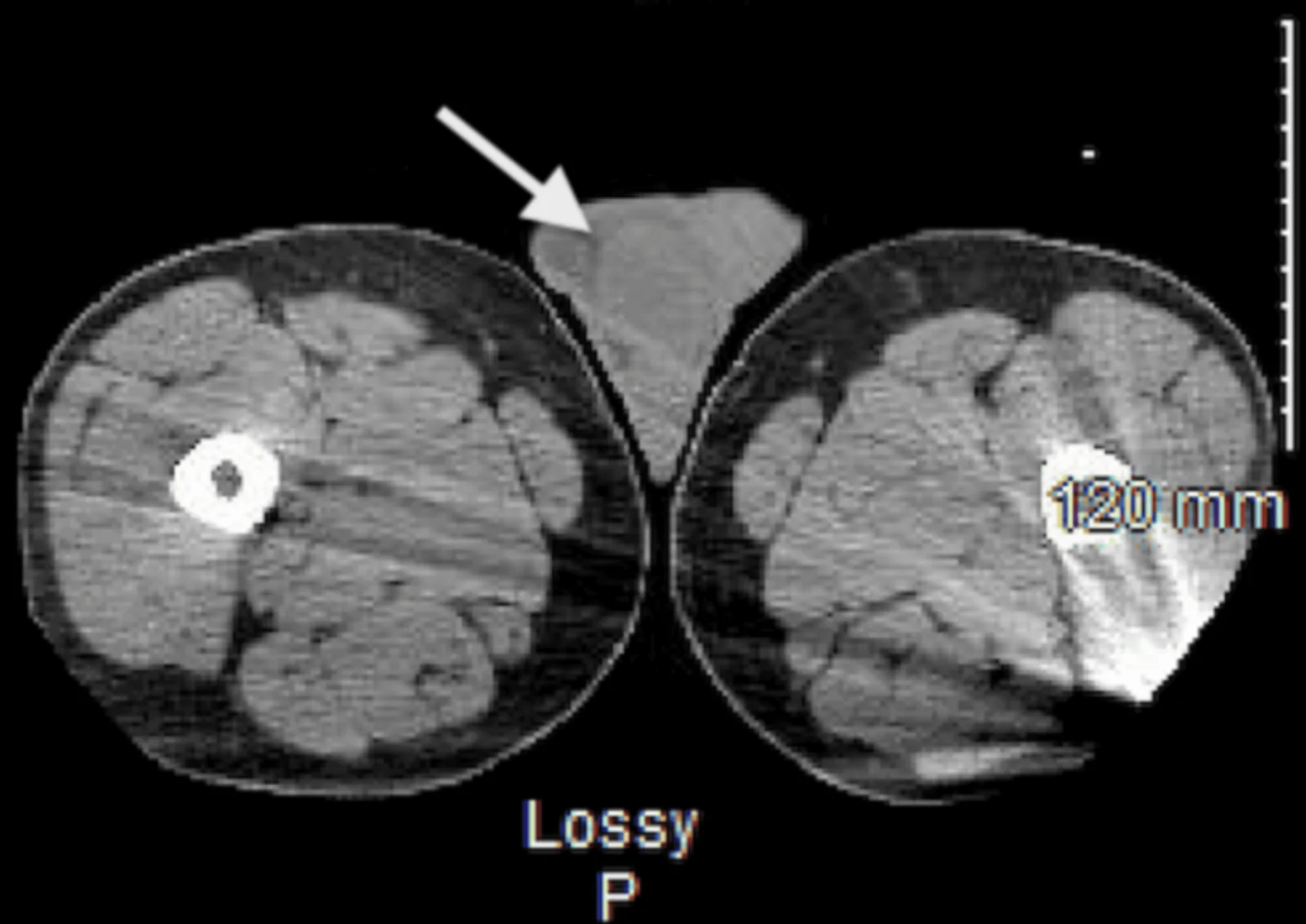
Inferior testicular displacement on preoperative imaging. CT demonstrates inferior displacement of the right testis within the right hemiscrotum (white arrow). Suprapubic testicular displacement was not present on this image and was identified only during surgery approximately 48 h later.

Computed tomography (CT) of the abdomen and pelvis was reviewed by the attending radiologist and surgical team. It demonstrated a midline/right paramedian ventral fascial defect measuring 8.7×10.9 cm in transverse and craniocaudal dimensions, with a hernia sac measuring approximately 28.6×16.4×14.8 cm and containing omentum and small-bowel loops (Figure 1). A separate right indirect inguinal defect measured approximately 4 cm. The ventral hernia extended inferiorly along the right lower abdominal wall toward the right hemiscrotum (Figure 2). CT suggested extension along the right inguinal region but could not determine reliably whether the scrotal tract was within or superficial to the inguinal canal (Figure 3). The right testis was displaced inferiorly within the hemiscrotum and was not suprapubic on CT (Figure 4). Scrotal ultrasonography demonstrated preserved bilateral arterial and venous Doppler flow without torsion or acute ischemia. Additional findings included a simple right intratesticular cyst, an epididymal head cyst, mild bilateral varicoceles, and small bilateral hydroceles (Table 2).

**Table 2:** Scrotal ultrasonography findings.

Finding	Result
Right testicular perfusion	Preserved arterial and venous Doppler flow
Left testicular perfusion	Preserved arterial and venous Doppler flow
Acute testicular ischemia	Not identified
Testicular torsion	Not identified
Right intratesticular cyst	Simple cyst
Epididymal head cyst	Present
Varicoceles	Mild and bilateral
Hydroceles	Small and bilateral

Approximately 48 h after CT, the patient underwent diagnostic laparoscopy. Two separate defects were identified as follows: a large ventral/incisional defect corresponding to the radiographic 8.7×10.9 cm defect and a right indirect inguinal defect measuring approximately 4 cm. Dense adhesions and distorted anatomy prevented safe laparoscopic dissection, prompting conversion to an open abdominal approach.

Open exploration demonstrated that the scrotal component originated from the ventral/incisional hernia and coursed through a subcutaneous plane superficial to the inguinal canal before entering the right hemiscrotum. The right testis was found in a suprapubic position, despite having been inferiorly displaced within the hemiscrotum on imaging 48 h earlier. The record establishes an interval change in position but does not establish its mechanism. Traction from the hernia sac, patient movement, or manipulation between imaging and surgery were considered possible explanations; attempted self-reduction was not documented and not assumed.

The hernia sac was densely adherent to the spermatic cord and right testis, requiring difficult adhesiolysis. The vas deferens, epididymis, testicular artery, pampiniform venous plexus, and associated cord structures were individually identified and preserved. The small bowel was viable and was reduced without resection. The operative record documented an open onlay ventral repair using a 12×12 cm macroporous polypropylene mesh; the exact circumferential overlap was not recorded. The separate indirect inguinal defect was repaired using a Bassini tissue repair without additional synthetic mesh. Total operative duration was 45 min, estimated blood loss was 200 mL, and no injury to bowel, vas deferens, epididymis, or testicular vessels was documented. Intraoperative photographs were not obtained (Table 3). The right testis was returned to a dependent scrotal position. The postoperative course was uncomplicated, and the patient was discharged on postoperative day two. He did not attend scheduled follow-up, and postoperative scrotal Doppler ultrasonography was therefore not obtained (Table 4).

**Table 3:** Operative findings and repair.

Domain	Finding
Ventral/incisional defect	Radiographic size 8.7×10.9 cm; bowel- and omentum-containing
Hernia sac	Approximately 28.6×16.4×14.8 cm on CT
Separate inguinal defect	Right indirect defect, approximately 4 cm
Aberrant route	Subcutaneous plane superficial to the inguinal canal
Bowel viability	Viable; reduced without resection
Testicular position	Suprapubic intraoperatively; restored to dependent scrotal position
Cord structures	Vas deferens, epididymis, testicular artery, pampiniform plexus, and associated structures preserved
Ventral repair	Open onlay repair with macroporous polypropylene mesh documented as 12×12 cm
Inguinal repair	Bassini tissue repair without separate synthetic mesh
Operative duration/blood loss	45 min/200 mL
Intraoperative photographs	Not obtained

**Table 4:** Chronological timeline of the patient's clinical course.

Time point	Clinical event
Eight years earlier	Laparoscopic right inguinal hernia repair for a direct inguinal hernia
Months before presentation	Recurrent right groin bulging with spontaneous reduction
Week before presentation	Increasing abdominal and groin pain and swelling
Hospital day 0	Emergency presentation with painful, persistently non-reducible ventral and inguinoscrotal swelling
Hospital day 0	CT demonstrated an incarcerated ventral hernia extending toward the right hemiscrotum and inferior displacement of the right testis
Hospital day 0-1	Scrotal Doppler ultrasonography demonstrated preserved testicular perfusion
Hospital day 2	Diagnostic laparoscopy converted to open exploration
Operation	Separate ventral/incisional and indirect inguinal defects identified; ventral sac tracked superficial to the inguinal canal; right testis found suprapubic
Postoperative day 2	Discharged after uncomplicated recovery

## Discussion

The principal novelty of this case is the route taken by the scrotal component of the ventral incisional hernia. Standard indirect scrotal hernias pass through the deep inguinal ring, inguinal canal, and superficial ring. In contrast, the ventral sac in this patient traveled through subcutaneous abdominal-wall tissues superficial to the canal before becoming densely adherent to the spermatic cord and testis. This route altered the expected operative landmarks and exposed both superficial abdominal-wall structures and the spermatic cord to injury.

A focused literature review identified multiple reports of giant inguinoscrotal hernias and one incisional hernia containing a bladder diverticulum that extended to the base of the scrotum [8,9]. The latter demonstrates that incisional defects can rarely communicate with the scrotal region, but it differs substantially from this case in content, route, and operative anatomy. We found no closely analogous bowel-containing midline incisional hernia following a subcutaneous plane superficial to the inguinal canal with an interval change in testicular position. The present configuration is therefore best described as apparently rare rather than unprecedented.

The prior laparoscopic inguinal repair is relevant because trocar-site incisional hernia is a recognized delayed complication of laparoscopic surgery [10]. However, the original operative report did not provide sufficient detail to confirm that the current ventral defect arose from a specific trocar site, and the manuscript does not infer that relationship as fact. Similarly, severe heart failure was clinically important for anesthetic risk and physiologic reserve, but available evidence does not establish it as the cause of the hernia or its unusual trajectory. Congestive heart failure has been associated with increased perioperative risk in ventral hernia repair and is therefore best treated as a risk modifier rather than a mechanistic explanation [11].

The discordance between imaging and operative testicular position also requires cautious interpretation. CT showed inferior displacement within the hemiscrotum, whereas the testis was suprapubic at surgery 48 h later. This establishes dynamic positional change but does not prove why it occurred. Traction from the hernia sac, patient movement, or manipulation are plausible, but none can be confirmed from the record. Explicit separation of observed findings from proposed mechanisms strengthens the clinical interpretation.

The anatomical structures at risk differed from those in a standard inguinal repair. Conventional inguinal dissection places the vas deferens, testicular vessels, pampiniform plexus, genital branch of the genitofemoral nerve, ilioinguinal nerve, iliohypogastric nerve, and inferior epigastric vessels at risk [6,12]. In this case, the superficial tract additionally traversed Scarpa fascia and the territory of the superficial epigastric and superficial circumflex iliac vessels, abdominal-wall sensory nerves, and subcutaneous vascularity before reaching the scrotum. Dense adherence to the testis and cord then recreated the familiar risks of vascular compromise, vasal injury, postoperative ischemia, hydrocele, atrophy, infertility, and chronic orchialgia [6,12].

CT was valuable for defining the fascial defect, sac dimensions, bowel content, and testicular displacement, but it did not accurately resolve the final relationship of the tract to the inguinal canal. The operative findings demonstrate why imaging should guide rather than dictate the surgical approach in markedly distorted anatomy [7]. Scrotal Doppler ultrasonography was also important because it established preserved preoperative testicular perfusion despite the inability to palpate the right testis separately.

The clinical findings supported incarceration as follows: acute worsening pain, persistent non-reducibility, nausea and emesis, enlargement of a previously reducible bulge, and bowel within the sac. The normal lactate and absence of peritonitis reduced concern for advanced bowel necrosis but did not exclude evolving strangulation, making timely exploration appropriate. The viable bowel avoided resection and permitted prosthetic reinforcement of the ventral repair.

The report documents the repair performed but does not propose it as a general standard. Contemporary groin hernia guidance favors mesh-based repair for most adults and recommends the Shouldice technique when a non-mesh approach is selected [13]. A Bassini tissue repair was used for the separate indirect defect in this complex operation. The operative record documented a 12×12 cm onlay mesh for the ventral defect, but exact mesh overlap was not recorded. Because the patient did not attend follow-up, the report cannot assess long-term recurrence, chronic groin pain, or delayed testicular complications. Additional limitations include incomplete details from the original operation, absence of intraoperative photographs, and lack of postoperative Doppler assessment.

## Conclusions

This case demonstrates an apparently rare bowel-containing ventral incisional hernia that extended into the right hemiscrotum through a subcutaneous plane superficial to the inguinal canal. Preoperative CT and scrotal Doppler ultrasonography defined the defect, contents, and preserved testicular perfusion, but operative exploration was required to reveal the true trajectory and suprapubic position of the right testis.

When ventral, inguinal, and scrotal anatomy are simultaneously distorted, surgeons should anticipate departure from standard planes and protect both superficial abdominal-wall structures and the vas deferens, epididymis, testicular vessels, pampiniform plexus, and associated nerves. Careful distinction between documented findings and proposed mechanisms is essential when imaging and operative anatomy differ.
